# The Press and the Hospitals

**Published:** 1890-12-13

**Authors:** 


### The Hospital

A Weekly Journal of

Science, Medicine, Nursing, and Philanthropy.

*For Hospitals, Asylums, Infirmaries, Dispensaries, Medical Practitioners, Students, Nurses, and the Charitable Public.*

Vol. IX.—No. 220.] [December 13, 1890.

### The Press and the Hospitals.

Voluntary Hospitals live by popular favour, and to take away that is to deprive them of the breath of life. The newspapers of this country possess great, and on the whole, well deserved influence. Although no newspaper, or combination of newspapers, can kill a good and strong cause, yet they can so far strangle even deserving institutions as to make their lives feeble and practically useless. With some diffidence as to ourselves, but with much confidence and courage as to our cause, we venture to stand in the breach on behalf of the London Hospital, and to make the best effort which may be possible to put all pressmen in possession of the actual facts and state of the case. We are impelled to this course by the attitude of the *Pall Mall Gazette* during the recent controversy. If the *Pall Mall* had been an obscure print, destitute of reputation and public influence, a wise discretion might have left it alone. But a succession of able, honest, and eminent editors; and an unfaltering, if sometimes mistaken devotion to the cause of the downtrodden and oppressed, entitle that paper to the respect of all fair-minded men, and make its conversion to juster convictions, a work eminently desirable.

Mrs. Robert Hunter has championed the assailants of the London Hospital, and has herself been championed by the *Pall Mall Gazette*. So far as we know no other paper of standing has taken that side of the controversy. This of itself is a significant fact in fair-minded England. We are desirous of doing full justice to Mrs. Hunter. At the beginning of this controversy, we understand, she was confined to her house by illness. It was when she was thus lying ill, that a story of supposed wrongs endured by probationers and other nurses at the London Hospital was poured into her ear. Burning with a desire to redress those wrongs, she sought help among matrons and nurses; got the case brought before the Lords' Committee; secured the sympathy and help of the *Pall Mall Gazette*; and finally formulated a series of charges, and laid them before the managers of the London Hospital. What we desire now to do, is to show to the public and the lay press the exact value of those charges; and also to make it plain that the *Pall Mall Gazette*, in giving to Mrs. Hunter its undiscriminating support, has either been misled, or has wilfully elected to follow a wrongful course to the injury of London's greatest and most indispensable medical charity.

On the fourteenth of October last, and in consequence of the charges that had been made by Mrs. Hunter and others, a sub-committee was appointed by the governors, "to report to the House Committee on the allegations which have recently been made against the nursing department." The report of the sub-committee, containing the several allegations, with detailed replies thereto, will be found at page liv. of the "Nursing Supplement" in our present issue. The charges made were five in number; and briefly summarised are these: That the matron has too much power at the London Hospital; that insufficiently-trained nurses are sent out to private patients; that the nurses' food is insufficient and unsuitable; that the staff is insufficient, the responsibilities of inexperienced nurses too great, and the paying probationers too numerous; and that, finally, too much menial work is cast upon the nurses, their hours are too long, and they are overworked. The replies of the sub-committee to these charges may be read in detail on pages liv., lv., and lvi. of the "Nursing Supplement." They are to this effect: That the matron of the London Hospital has exactly the amount of power recommended by Miss Nightingale for hospital matrons, and exercised by such matrons universally. The charge that defectively-trained nurses are sent to private patients is proved by documentary evidence to be unfounded. The third accusation, which states the food to be unsatisfactory, members of the Committee declare of their own knowledge to be groundless. Concerning the fourth charge, which relates to the responsibilities of half-trained nurses and the preponderance of paying probationers, the sub-committee are of opinion that the "grievances have no practical existence." The fifth and last of the allegations, that the nurses are overworked and have too long hours, are answered by the statement that in this respect the London challenges comparison, and compares favourably with any other hospital in the metropolis or the country. Concerning the matron, they report that she "possesses, and in the judgment of the Committee deserves, their entire confidence" . . . and they affirm that they "would regard it as in the highest degree ungrateful and unjust to ignore the great services which she has rendered to the hospital, or consent to any step which would imply a want of confidence in her, or tend to weaken her authority."

Now this report the Sub-Committee took nearly two months to prepare, and on Wednesday week it was presented to a meeting of the governors specially called to consider it. That meeting consisted of at least three hundred governors, whereas the usual number of governors attending a meeting is about thirty. Mrs. Hunter attended, together with her husband, Mr. Hunter, and Mr. Yatman, the father of the probationer who had had so much to do with originating the charges and allegations. The report of the Sub-Committee was read; Mrs. Hunter's animadversions were duly made, and the question was put to the vote. In all this large meeting, only four hands were held up against the adoption of the report; and of those four, one hand was that of Mrs. Hunter, another that of Mr. Hunter, and a third that of Mr. Yatman. To the best of our belief, those three persons were not ordinary governors of the London Hospital, but governors who had qualified themselves solely for the purpose of making and supporting the charges and allegations. Will any reasonable Englishman or Englishwoman believe for a moment that three persons who, up to the present year, had taken no interest whatever in the working of the London Hospital were right in their charges; and that three hundred, who were for the most part subscribers and governors of long-standing, were wrong? If the opinions of the three are to be accepted in preference to those of the better informed three hundred, public business in this country will be no longer possible.

In spite of these facts, the *Pall Mall Gazette* still professed to believe that the three were right and the three hundred wrong. But this is too ridiculous. The editor seems to have entirely abandoned his usual fairness on this subject. Indeed, we are bound to conclude that, for some reason or other, he has been determined that Mrs. Robert Hunter should be right though all the world were wrong. It was a case of Mrs. Hunter *contra mundum*. Can the editor of the *Pall Mall* say in his conscience that he has acted fairly and squarely in this matter? Has he availed himself to the full of the opportunities offered him by the hospital authorities for personally investigating the condition of the London Hospital, and for ascertaining for himself the truth or falsehood of Mrs. Hunter's charges? Has he published all kinds of statements made by anonymous persons on behalf of the assailants of the hospital, and has he refused to publish signed letters sent for insertion by the friends of the institution? Is he aware that the editorial notes which appeared at page 2 of the *Pall Mall* of December 4th, and which were couched in the most abusive language, were completely answered in every detail by an outline report of the Committee's proceedings at page 7 of the very same issue? If the editor of the *Pall Mall Gazette* cannot deny the truth of the allegations we are now making against him, what shall be said of his sense of public duty, or of his regard for that fairness and impartiality which Englishmen regard as their most distinguishing characteristic? Is it the fact that the editor of the *Pall Mall* has been willing to sacrifice the interests of the London Hospital, and with them the much more serious and pathetic interests of the poor of the East-end of London, for the sake of gratifying two or three, perhaps well-meaning, but certainly misguided, persons? We have no hesitation in saying here that the mildest opinion which can be expressed concerning the editor of the *Pall Mall* is that in acting as he has done he has committed an error of judgment of which he ought to be ashamed. If he be made of the same honourable stuff as his two predecessors, he will hasten with all speed to make the fullest possible amends for his conduct.

